# Draft Genome Sequence of Lactobacillus crispatus UMB1163, Isolated from the Female Urinary Tract

**DOI:** 10.1128/MRA.00404-20

**Published:** 2020-06-04

**Authors:** Fabeiha Khan, Taylor Miller-Ensminger, Adelina Voukadinova, Alan J. Wolfe, Catherine Putonti

**Affiliations:** a Department of Biology, Loyola University Chicago, Chicago, Illinois, USA; b Bioinformatics Program, Loyola University Chicago, Chicago, Illinois, USA; c Department of Microbiology and Immunology, Stritch School of Medicine, Loyola University Chicago, Maywood, Illinois, USA; d Department of Computer Science, Loyola University Chicago, Chicago, Illinois, USA; University of Rochester School of Medicine and Dentistry

## Abstract

Lactobacillus crispatus is a Gram-positive bacterium shown to protect against urinary and vaginal infections. Here, we report the draft genome sequence of L. crispatus UMB1163, isolated from the female urinary tract.

## ANNOUNCEMENT

Lactobacillus crispatus is a nonpathogenic bacterium native to the healthy female urogenital tract (1, 2). L. crispatus is critical in preventing common bacterial infections (3–5), such as bacterial vaginosis and vulvovaginal atrophy (VVA), by preserving low pH and producing hydrogen peroxide (4). L. crispatus creates a biofilm in the vaginal epithelium, providing protection against pathogens that cause sexually transmitted diseases and urinary tract infections. Due to its ability to limit pathogens in the urogenital system, L. crispatus strains are being explored for use as a probiotic to prevent urinary tract infections (UTI) in women (6). Here, we present the draft genome sequence of L. crispatus UMB1163, isolated from the bladder of a female with a UTI.

The urine specimen was collected via a transurethral catheter from a woman seeking clinical care at Loyola University Medical Center’s Female Pelvic Medicine and Reconstructive Surgery Center (Maywood, IL, USA) as part of a prior institutional review board (IRB)-approved study (Loyola University Chicago, IRB approval no. 206469) (7). L. crispatus was isolated from this urine specimen using the expanded quantitative urinary culture (EQUC) method (8). The genus and species of the bacterium were determined using matrix-assisted laser desorption ionization–time of flight (MALDI-TOF) mass spectrometry (8) prior to storage at −80°C. L. crispatus UMB1163 was streaked onto a Columbia nalidixic acid (CNA) agar plate and incubated for 24 h at 35°C with 5% CO_2_. A single colony was selected and incubated in De Man, Rogosa, and Sharpe (MRS) liquid medium supplemented with newborn calf serum (50 ml/liter) and incubated for 24 h at 35°C with 5% CO_2_. DNA was extracted using the Qiagen DNeasy blood and tissue kit with a Gram-positive protocol modified as follows: 230 μl of lysis buffer (180 μl of 20 mM Tris-Cl, 2 mM sodium EDTA, and 1.2% Triton X-100) and 50 μl of lysozyme were used in step 2, and the incubation time in step 5 was reduced to 10 min. The DNA was quantified using a Qubit fluorometer. Sequencing was done at the University of Pittsburgh Microbial Genomic Sequencing Center (MiGS) on the Illumina NextSeq 550 platform. MiGS created the libraries using the Illumina Nextera kit. Sequencing produced 1,781,766 pairs of 150-bp reads. Sickle v1.33 (https://github.com/najoshi/sickle) was used to trim the raw reads, which were then assembled using SPAdes v3.13.0 with the “only-assemble” option for k values of 55, 77, 99, and 127 (9). Genome coverage was calculated using BBMap v38.4 (https://sourceforge.net/projects/bbmap/). We used PATRIC v3.6.3 to annotate the genome (10). The publicly available genome was annotated with the NCBI Prokaryotic Genome Annotation Pipeline (PGAP) v4.11 (11). Unless otherwise stated, default parameters were used for each software tool.

The L. crispatus UMB1163 draft genome sequence is 2,384,113 bp long assembled into 151 contigs with an *N*_50_ score of 26,484 bp, genome coverage of 177×, and GC content of 36.6%. The L. crispatus assembly has 66 tRNAs and 5 complete rRNAs (3 5S, 1 16S, and 1 23S). PGAP identified 2,353 protein-coding genes. PATRIC identified 1 CRISPR array, with 7 spacer sequences. While numerous genomes of L. crispatus strains from the vaginal microbiome are available, a greater representation of the genetic diversity of this species within the urinary tract is needed. Sequencing isolates from the urinary tract will provide insight into the role that L. crispatus plays in the female urinary tract.

### Data availability.

This whole-genome shotgun project has been deposited in GenBank under the accession no. JAAUWJ000000000. The raw sequence reads were deposited in the SRA under the accession no. SRR11441017.

## References

[1] Lepargneur J-P 2016 Lactobacillus crispatus as biomarker of the healthy vaginal tract. Ann Biol Clin (Paris) 74:421–427. (In French.) doi:10.1684/abc.2016.1169. 27492695

[2] Pearce MM, Hilt EE, Rosenfeld AB, Zilliox MJ, Thomas-White K, Fok C, Kliethermes S, Schreckenberger PC, Brubaker L, Gai X, Wolfe AJ 2014 The female urinary microbiome: a comparison of women with and without urgency urinary incontinence. mBio 5:e01283-14. doi:10.1128/mBio.01283-14. 25006228 PMC4161260

[3] Brotman RM, Shardell MD, Gajer P, Fadrosh D, Chang K, Silver MI, Viscidi RP, Burke AE, Ravel J, Gravitt PE 2018 Association between the vaginal microbiota, menopause status, and signs of vulvovaginal atrophy. Menopause 25:1321–1330. doi:10.1097/GME.0000000000001236. 30358729

[4] Mitchell C, Manhart LE, Thomas K, Fiedler T, Fredricks DN, Marrazzo J 2012 Behavioral predictors of colonization with Lactobacillus crispatus or Lactobacillus jensenii after treatment for bacterial vaginosis: a cohort study. Infect Dis Obstet Gynecol 2012:706540. doi:10.1155/2012/706540. 22693410 PMC3369434

[5] Aroutcheva A, Gariti D, Simon M, Shott S, Faro J, Simoes JA, Gurguis A, Faro S 2001 Defense factors of vaginal lactobacilli. Am J Obstet Gynecol 185:375–379. doi:10.1067/mob.2001.115867. 11518895

[6] Li T, Liu Z, Zhang X, Chen X, Wang S 2019 Local probiotic Lactobacillus crispatus and Lactobacillus delbrueckii exhibit strong antifungal effects against vulvovaginal candidiasis in a rat model. Front Microbiol 10:1033. doi:10.3389/fmicb.2019.01033. 31139166 PMC6519388

[7] Price TK, Dune T, Hilt EE, Thomas-White KJ, Kliethermes S, Brincat C, Brubaker L, Wolfe AJ, Mueller ER, Schreckenberger PC 2016 The clinical urine culture: enhanced techniques improve detection of clinically relevant microorganisms. J Clin Microbiol 54:1216–1222. doi:10.1128/JCM.00044-16. 26962083 PMC4844725

[8] Hilt EE, McKinley K, Pearce MM, Rosenfeld AB, Zilliox MJ, Mueller ER, Brubaker L, Gai X, Wolfe AJ, Schreckenberger PC 2014 Urine is not sterile: use of enhanced urine culture techniques to detect resident bacterial flora in the adult female bladder. J Clin Microbiol 52:871–876. doi:10.1128/JCM.02876-13. 24371246 PMC3957746

[9] Bankevich A, Nurk S, Antipov D, Gurevich AA, Dvorkin M, Kulikov AS, Lesin VM, Nikolenko SI, Pham S, Prjibelski AD, Pyshkin AV, Sirotkin AV, Vyahhi N, Tesler G, Alekseyev MA, Pevzner PA 2012 SPAdes: a new genome assembly algorithm and its applications to single-cell sequencing. J Comput Biol 19:455–477. doi:10.1089/cmb.2012.0021. 22506599 PMC3342519

[10] Brettin T, Davis JJ, Disz T, Edwards RA, Gerdes S, Olsen GJ, Olson R, Overbeek R, Parrello B, Pusch GD, Shukla M, Thomason JA, Stevens R, Vonstein V, Wattam AR, Xia F 2015 RASTtk: a modular and extensible implementation of the RAST algorithm for building custom annotation pipelines and annotating batches of genomes. Sci Rep 5:8365. doi:10.1038/srep08365. 25666585 PMC4322359

[11] Tatusova T, DiCuccio M, Badretdin A, Chetvernin V, Nawrocki EP, Zaslavsky L, Lomsadze A, Pruitt KD, Borodovsky M, Ostell J 2016 NCBI Prokaryotic Genome Annotation Pipeline. Nucleic Acids Res 44:6614–6624. doi:10.1093/nar/gkw569. 27342282 PMC5001611

